# Operating Mechanism Principles and Advancements for Halide Perovskite-Based Memristors and Neuromorphic Devices

**DOI:** 10.1021/acs.jpclett.4c02170

**Published:** 2024-09-27

**Authors:** So-Yeon Kim, Heyi Zhang, Jenifer Rubio-Magnieto

**Affiliations:** † Instituto de Tecnología Química (ITQ), Universitat Politècnica de València- Consejo Superior de Investigaciones Científicas (UPV-CSIC), 46022 València, Spain; ‡ Institute of Advanced Materials (INAM), Universitat Jaume I, 12006 Castelló, Spain

## Abstract

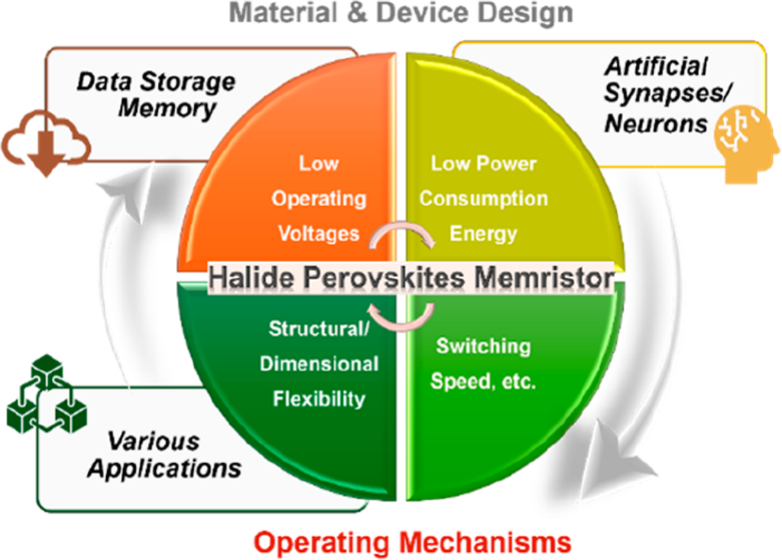

With the advent of the generation of artificial intelligence (AI) based on big data-processing technologies, next-generation memristor and memristive neuromorphic devices have been actively studied with great interest to overcome the von Neumann bottleneck limits. Among various candidates, halide perovskites (HPs) have been in the spotlight as potential candidates for these devices due to their unique switching characteristics with low energy consumption and flexible integration compatibility across various sources for scalability. We outline the characteristics and operating principles of HP-based memristors and their neuromorphic devices. We explain filamentary- and interface-type switching according to the type of conducting pathway occurring inside the active HP layer and the operating mechanisms depending on the species that make up this conducting pathway. We summarize the types and mechanisms of current changes beneficial for neuromorphic device applications and finally organize various suggested analysis tools and physical models to enable experimental determination of switching mechanisms from various perspectives.

The memristor (memory + resistor), also called resistive random-access memory (ReRAM), has received much attention as both next-generation memory and bioinspired neuromorphic devices over conventional NAND flash memory because it overcame the scaling and data transfer rate limits related to Moore’s law and the von Neumann bottleneck.^1,2^ In particular, halide perovskite (HP)-based memristive devices show excellent characteristics of ultralow power consumption with ionic–electronic complex driving motion. It has now arrived at a stage where it is necessary to organize existing memristive operation mechanisms that have been spread too widely and present the developmental direction to realize the multifunctional applications of HP-based memristors and bioinspired memristive devices.^3,4^ We here briefly introduce HP-based memristive devices (involving memory, artificial neurons/synapses, bioinspired devices, etc.) and focus on the various operation mechanisms that have been reported so far and analysis tools and efforts that have been tried to prove them. This Mini-Review aims to be a guide for future HP-based memristive research by suggesting the importance of analyzing the operation mechanism principles.

Faced with the recent generation of artificial intelligence (AI) and the metaverse demanding vast amounts of data-processing technologies, the memristor also has been in the spotlight in both high-capacity memory devices and memristive neuromorphic computing systems that mimic the human brain, using the resistance (conductance) change of the memristor with the satisfactory characteristics such as high integration density, low power consumption, flexibility, etc.^5−8^ To obtain such superior performance and expand appropriate applications (memory, artificial neuron, artificial synapse, etc.), it is necessary to understand the operation mechanism principle of the memristor and select proper electrodes and active switching layer, because the electrical and physical properties of all components affect the driving characteristics.

There are many candidates for the active switching layer of memristors and neuromorphic devices, such as oxides, chalcogenides, organic semiconductors, graphene, perovskites, etc. Among them, halide perovskites (HPs), which have been particularly in the spotlight in the solar cell field, are also a focus for memristor research using their characteristic current–voltage (*I*–*V*) hysteresis with unique optoelectronic properties such as long charge carrier diffusion length, photoactivity, bandgap tunability, easy fabrication, flexibility, and so on.^9,10^ HPs show memristive operation by various external stimuli, such as voltage stimuli, photonic illumination, etc.,^11,12^ with various performances and conduction mechanisms in memristors and their neuromorphic computing systems because their various derivatives each show unique bandgap and *I*–*V* hysteresis upon varying the composition and ratio of each site in a typical ABX_3_ structure [A = monovalent organic/inorganic cation; B = central metallic cation (e.g., Pb, Sn, etc.); and X = halide anion (e.g., I, Br, Cl)].

However, it is also true that HPs show rather complex ionic/electronic migration in the memristors, so insight and research on the memristive mechanisms are insufficient.^13−15^ Aside from reporting high performance and various HPs for memristors, understanding the memristive mechanisms and physical circuit systems is one of the most important stepping stones to obtaining higher performance and practical operations. Here, we focus on explaining advancements to understand operating mechanism principles and physical circuit models/proposals but also provide a brief and basic introduction to HP-based memristors and neuromorphic devices as well as insights and directions for future memristor research.

## 1 Halide Perovskite-Based Memristors and Neuromorphic Devices

The human brain is composed of 100 billion neurons and 100 trillion synapses, which recognize and transmit electrical or chemical signals to another nerve cell (presynapse to postsynapse) and process huge amounts of information with low energy consumption and fast speed (femtoseconds).^16^ Much research is focused on mimicking the advanced neuronal–synaptic circuit system of the human brain, called neuromorphic computing systems, to overcome scaling limits such as the von Neumann bottleneck seen in existing conventional computing systems.^17^ The key point of these applications is to modulate the memristive devices’ physical structure and the characteristics of the active resistive switching (RS) layer in the simple sandwiched device composed mainly of metal electrodes and an active switching layer. It should store and process the available information without unintended motion, thereby obtaining appropriate performance parameters for each memristive device. Therefore, in this section, after introducing the basic parameter definitions of memristors and neuromorphic devices, HP-based memristive devices will be explained.

### 1.1 Key Parameters of Memristors

Bipolar RS memories run back and forth between the high resistance state (HRS) and low resistance state (LRS); the change in conductance from HRS to LRS is called the SET process, and the opposite change is called the RESET process by an external applied stimulus such as electric field, photonic illumination, etc.^11,12^ In addition, sometimes for stable repetitive switching, a step that requires a higher electric field than the SET process in the first switching sweep is required, which is defined as a forming process (electroforming step). Switching voltages refer to the voltages at the points at which the SET/RESET processes occur. ON/OFF ratio is the resistance (current) gap between HRS and LRS read at a specific voltage, and the speed at which the SET and RESET processes occur is called switching speed, which are the elements representing high-capacity and high-speed memristive devices, respectively. Compliance current is a current limit that prevents excessive current flow to the device at once or for multilevel storage. Endurance and retention time are device durability-related factors that indicate how many times the devices can run and how long they can maintain resistance values, respectively. In addition, memristors are also evaluated for mechanical–electrical stability measured in air or heat exposure, scalability according to the stacking ability in the crossbar array, and integration ability with other sources for integrated circuits.

### 1.2 Key Elements of Neuromorphic Devices

To determine if the memristors can be extended to neuromorphic memristive computing systems, they should not only exhibit the above-mentioned various memristive characteristics but also be evaluated for linearity, *I*–*V* symmetry, energy power consumption, and so on. With these characteristics, investigations such as excitatory/inhibitory postsynaptic current (EPSC/IPSC), short-term/long-term plasticity (STP/LTP) transition, paired-pulse facilitation (PPF), and spike-dependent plasticity for various factors related to synaptic plasticity indicating the connection strength between neurons are conducted, where the memristors can play various roles such as neuron, synapse, long-term memory, and short-term memory depending on their characteristics.^4,5,8^ High linearity and symmetry are the basic needs for implementing an ideal neuromorphic computing system, which means high accuracy with minimal errors for updating weights of information in analogue conductance changes (synaptic weight). In artificial neural network systems (ANNs), an input spike signal is transmitted through a small gap between the cell membranes called the synaptic cleft from presynaptic neuron to postsynaptic neuron, and EPSC or IPSC is derived as the output current signals from the receptor of the postsynaptic neuron. Excitatory neurotransmitters activate the receptors in the postsynapse membrane and cause depolarization to enhance the action potential effect, while inhibitory neurotransmitters cause hyperpolarization to block the action potential. These neurotransmitters are controlled by external electrical stimulation (pulse), such as gate voltage, light sources, etc., in HP-based memristors. Synaptic plasticity, which represents information transfer in these neural network systems along with strengthening/weakening synaptic weight, can be largely divided into short-term potentiation/depression and long-term potentiation/depression depending on the memory duration time. After pulse stimuli, the conductance change in the active channel returns to its original state quickly in short-term plasticity (STP) and remains with the changed state in long-term plasticity (LTP), and when repeatedly encoded as STP, it can be converted to LTP that follows Hebbian learning. Paired-pulse facilitation (PPF) is one of the STP characteristics, which means the relative difference between two paired synaptic spike signals. Since the PPF ratio is greatly affected by the time interval between two pulses and even by external stimuli sources, it is important to control the accumulation time and stimuli methods in the memristive neuromorphic systems in terms of the conductive ions/species. Memristive neuromorphic systems with spiking neural network system (SNNs) are affected by the pulse width, interval, frequency, rate, etc. of the spike pulse transmitted between two neurons, followed by the close investigations such as spike-rate-dependent plasticity (SRDP), spike-timing-dependent plasticity (STDP), etc.

### 1.3 HP-Based Memristors and Neuromorphic Devices

**Figure 1:**
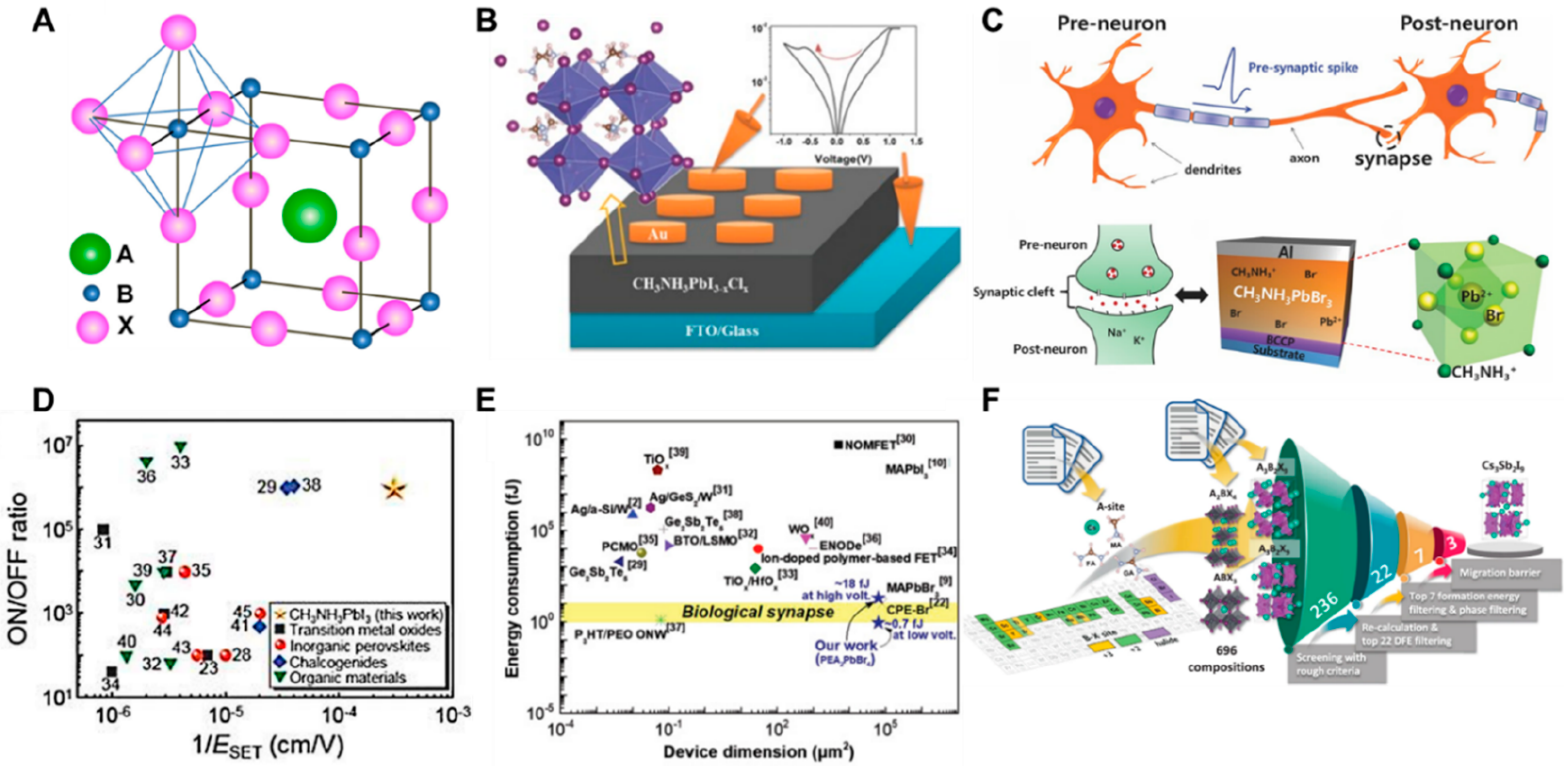
(A) Schematic of typical HP crystal structure. Reproduced from Park, N.-G. Perovskite Solar Cells: An Emerging Photovoltaic Technology. *Mater*. *Today***2015**, 18, 65.^18^ Copyright 2015 Elsevier. (B) *I*–*V* resistive switching behavior with schematic resistive switching device of Ag/CH_3_NH_3_PbI_3-*x*_Cl_*x*_/FTO structure. Reproduced from Yoo, E. J.; Lyu, M.; Yun, J. H.; Kang, C. J.; Choi, Y. J.; Wang, L. Resistive Switching Behavior in Organic–Inorganic Hybrid CH_3_NH_3_PbI_3–X_Cl_X_ Perovskite for Resistive Random Access Memory Devices. *Adv*. *Mater*. **2015**, 27, 6170.^19^ Copyright 2015 John Wiley and Sons. (C) Schematics of artificial synapses and device structures of Al/MAPbBr_3_/BCCP/ITO/Glass. Reproduced from Xu, W.; Cho, H.; Kim, Y. H.; Kim, Y. T.; Wolf, C.; Park, C. G.; Lee, T. W. Organometal Halide Perovskite Artificial Synapses. *Adv*. *Mater*. **2016**, 28, 5916.^20^ Copyright 2016 John Wiley and Sons. (D) Performance comparison indicators of HP with various candidates for resistive switching devices. Reproduced from Choi, J.; Park, S.; Lee, J.; Hong, K.; Kim, D.-H.; Moon, C. W.; Park, G. D.; Suh, J.; Hwang, J.; Kim, S. Y.; Jung, H. S.; Park, N.-G.; Han, S.; Nam, K. T.; Jang, H. W. Organolead Halide Perovskites for Low Operating Voltage Multilevel Resistive Switching. *Adv*. *Mater*. **2016**, 28 (31), 6562.^21^ Copyright 2016 John Wiley and Sons. (E) Performance comparison indicators of HP with various candidates for memristive neuromorphic devices. Reproduced from Kim, S.-I.; Lee, Y.; Park, M.-H.; Go, G.-T.; Kim, Y.-H.; Xu, W.; Lee, H.-D.; Kim, H.; Seo, D.-G.; Lee, W.; Lee, T.-W. Dimensionality Dependent Plasticity in Halide Perovskite Artificial Synapses for Neuromorphic Computing. *Adv*. *Electron*. *Mater*. **2019**, 5 (9), 1900008. Copyright 2019 John Wiley and Sons.^22^ (F) Schematic diagram showing the possibility of producing various participants through various combinations of HPs through compositional/structural engineering. Reproduced from Park, Y.; Kim, S. H.; Lee, D.; Lee, J. S. Designing Zero-Dimensional Dimer-Type All-Inorganic Perovskites for Ultra-Fast Switching Memory. *Nat*. *Commun*. **2021**, 12, 3527.^23^ Copyright 2021 Springer Nature.

Among various memristive candidates exhibiting resistive switching (RS) properties, such as oxides, graphene, two-dimensional (2D) materials, perovskites, etc., there has been much active research on HP-based memristors and neuromorphic devices with their characteristic *I*–*V* hysteresis, which is a loss-making disadvantage for other areas such as solar cells^18^ but is advantageous for memory effect (the typical HP crystal structure is described in Figure 1A).^24−26^ Xiao et al. first reported on switchable *I*–*V* hysteresis induced by ion drift in an HP layer, and a CH_3_NH_3_PbI_3–*x*_Cl_*x*_-based memristor was subsequently reported with bipolar resistive switching behaviors in 2015 by Yoo et al. (Figure 1B).^19,27^ The Au/MAPbI_3–*x*_Cl_*x*_/FTO device showed *I*–*V* hysteresis with low operating voltages (less than ∼1 V), where the RS phenomenon of the mixed organic–inorganic HP-based memristor system was explained by *I*–*V* log–log scale analysis and photoluminescence (PL) measurement: the current flows through a trap-controlled space-charge-limited conduction (SCLC) mechanism by halide vacancies/defects. Subsequently, a CH_3_NH_3_PbBr_3_-based two-terminal artificial synapse was first attempted in 2016 by Lee’s group (Figure 1C).^20^ MAPbBr_3_-based memristor, which was first applied as a neuromorphic device, successfully implemented artificial synaptic properties such as PPF, STDP, etc., depending on the number and amplitude of electrical pulses, which is driven by conductance changes because of ion vacancy defects across the HP-layer. This phenomenon was analyzed by Br element mapping before and after electrical pulse for LTP through transmission electron microscopy (TEM). In addition to memristive devices driven by these electrical stimuli, photonic memory and neuromorphic devices using HP’s photoelectrical properties have also been reported.^11,12,28,29^ The photonic stimuli alone or synergetic with voltage stimuli make the memristive devices drive along with the change of their internal resistance states. Guan et al. proposed the artificial iconic memories called photomemories, which is combined with resistive switching memory and photodetector functionalities of HP.^11^ The monolithic artificial iconic memory device is operated by storing the photosensing data. Liu et al. proposed an HP-based optical neuromorphic computing synapse with excellent photoresponsivity performance, enabling EPSC, PPF, SRDP, and other biological behaviors.^29^ Using these attempts as a stepping stone, an explosive number of HP-based memristive devices operated with various optoelectronic stimuli have been reported.

In addition to the most typical 3D ABX_3_ structure (A = monovalent organic or inorganic cation, B = divalent metal cation (Pb, Sn, etc.), X = halide anion), HPs possess a variety of lattice-dimensional/compositional tunability from 3D to low-dimensional 0D, 1D, and 2D structures and lead-less or lead-free HPs, depending on the ion species or ratio of each site.^22,23,30−33^ With these advantages, various candidates have been used in applications of HP-based memristors and successfully reported with unique RS characteristics (Figure 1D–F).^21−23^ The various mechanisms and changes in RS performance due to compositional and dimensional engineering are explained in the text. In Figure 1D–F, HPs show superior memristive properties in comparison to other materials, such as low operating voltages (even less 0.1 V), low power consumption energy (∼few femtojoules), fast switching speed (∼few tens of nanoseconds), large ON/OFF ratio (in particular, low-dimensional HPs show over 10^8^), flexibility, etc.^22,34−37^ When expanding their applications, each of these memristive properties is tailored to a purpose, such as memory device, bioinspired device, integrated chips, etc., which is the key to bring HP-based memristors to multifunctional devices. Nevertheless, the flow of conduction in unintended directions in HP-based memristive devices is still delaying the actual implementation, such as insufficient endurance, the fluctuations of multilevel resistance states, and sneak-current issues. Therefore, identifying the operating mechanism of the HP-memristor is a priority to properly modulate the current flows within these devices.

## 2 Operating Mechanism Principles and Analysis for HP-Based Memristive Devices

HP-based memristors show excellent characteristics in various fields, including memory, neuromorphic devices, and so on, but the unpredictable resistive randomness still holds applications back.^38^ Randomness caused by nonuniform distribution of defects or unreliable repeatability of the localized conducting pathway in the HP active layer results in low operating sustainability in memristors, especially nonlinear and asymmetric conductance changes in neuromorphic computing circuits.^39^ In addition, synaptic plasticity, which means conductivity change in memristors, is highly influenced by efficient brain mimic systems, depending on how they can respond to electrical signals through various physical and chemical mechanisms. Accordingly, a variety of electrical and mechanical efforts have been proposed to reduce this randomness in HP-based memristors. However, prior to these, the most important thing is to accurately identify and control the operating mechanism principles for solving these problems and for higher performance and stable device implementation. Therefore, from now on, after explaining the basic mechanism of HP-based memristive devices, the mechanism analysis and control efforts will be outlined.

Memristors are composed of simple metal–insulator–metal (MIM) structures, but various mechanisms and switching properties can be produced depending on the components and structures of the device, such as the physical-electrical-chemical properties of the switching active layer and the electrode types, etc. Therefore, a close understanding of the memristive driving principles changing the internal materials or external circuits of the device can cause a great deal of development in the memristors and their neuromorphic computing systems that require high accuracy and stability.

### 2.1 Filamentary-Type and Interface-Type Switching

**Figure 2:**
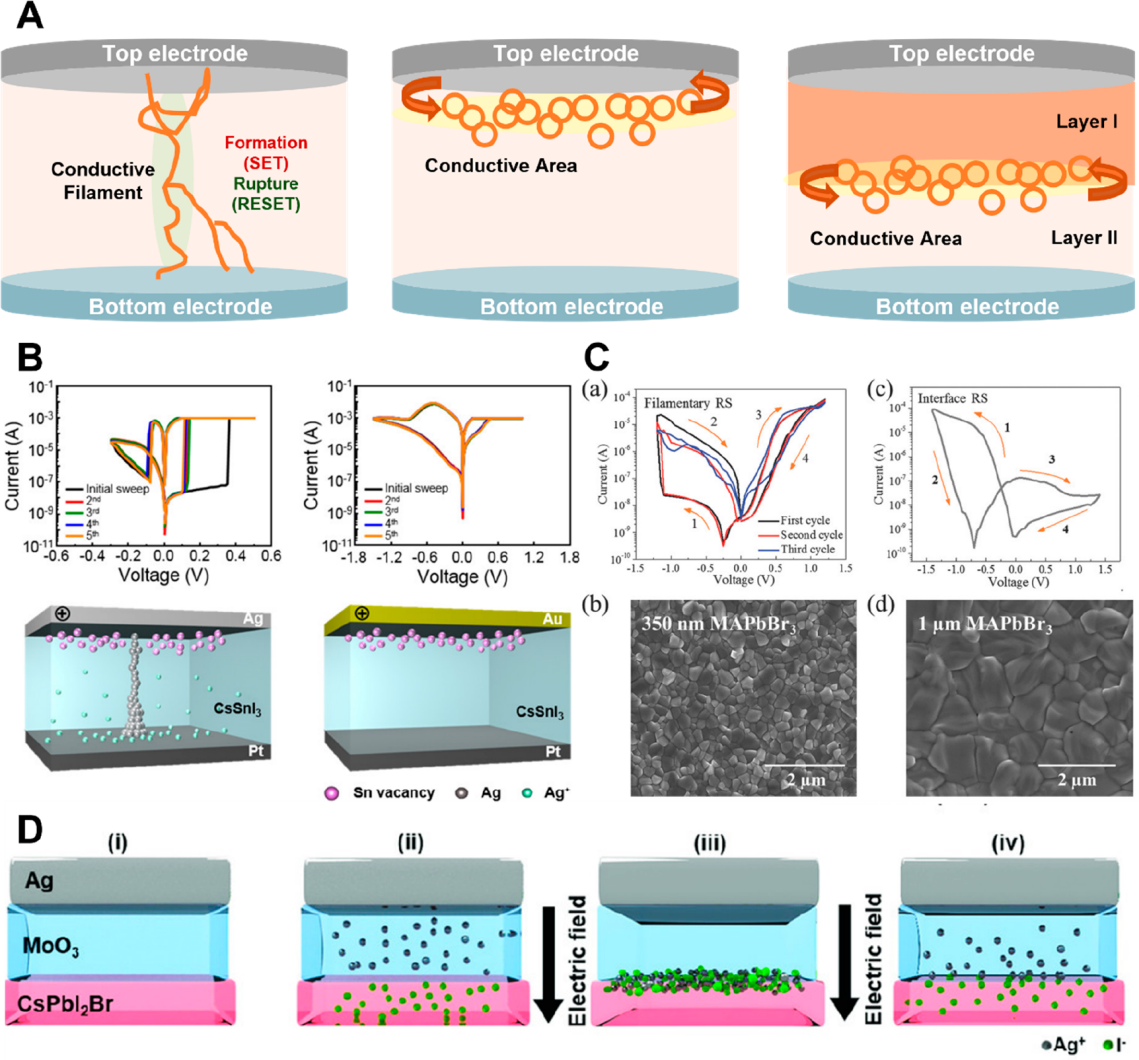
(A) Schematics for filamentary-type switching and interface-type switching. (B) Different *I*–*V* sweeps with schematic switching types between filamentary-type and interface-type switching behaviors. Reproduced from Han, J. S.; Le, Q. V.; Choi, J.; Kim, H.; Kim, S. G.; Hong, K.; Moon, C. W.; Kim, T. L.; Kim, S. Y.; Jang, H. W. Lead-Free All-Inorganic Cesium Tin Iodide Perovskite for Filamentary and Interface-Type Resistive Switching toward Environment-Friendly and Temperature-Tolerant Nonvolatile Memories. *ACS Appl*. *Mater*. *Interfaces***2019**, 11, 8155.^40^ Copyright 2019 American Chemical Society. (C) Different *I*–*V* switching loops and scanning electron microscopy (SEM) images of a thinner MAPbBr_3_-based memristor (filamentary-type) and a thicker MAPbBr_3_-based memristor (interface-type). Reproduced from Guan, X.; Hu, W.; Haque, M. A.; Wei, N.; Liu, Z.; Chen, A.; Wu, T. Light-Responsive Ion-Redistribution-Induced Resistive Switching in Hybrid Perovskite Schottky Junctions. *Adv. Funct*. *Mater*. **2018**, 28, 1704665.^41^ Copyright 2017 John Wiley and Sons. (D) Schematic interface-type switching operating processes at the hybrid interfaces between HP layer and MoO_3_ layer of Ag/MoO_3_/CsPbI_2_Br/MoO_3_/ITO device. Reproduced from Chen, L.-W.; Wang, W.-C.; Ko, S.-H.; Chen, C.-Y.; Hsu, C.-T.; Chiao, F.-C.; Chen, T.-W.; Wu, K.-C.; Lin, H.-W. Highly Uniform All-Vacuum-Deposited Inorganic Perovskite Artificial Synapses for Reservoir Computing. *Adv*. *Intell*. *Syst.***2021**, 3 (1), 2000196.^42^ Copyright 2021 John Wiley and Sons.

Depending on the types of internal resistance change within the active switching layer of the device, which is the core of the memristor driving, the switching type is largely reported as filamentary-type and interface-type.^40−43^ For each switching type, different electrical trends, such as the slope of the resistance change in the *I*–*V* curve and electrode/device structure dependence, appear in Figure 2.

In the case of the device switching to a filamentary-type, the conductive filament (CF) localized in the switching medium is formed and current changes flow through that (Figure 2A). Memristors flowing through the filamentary-type switching mainly show the conductance changes at the point where the filament is formed; that is, this type of memristor is driven by repeatedly undergoing the SET processes in which the CF is formed by the electric field and RESET processes in which the CF is partially or completely ruptured in the opposite electric field. In addition, as these localized CF thicknesses vary by controlling several factors such as compliance currents,^44^ applied voltages,^12^ and so on, the memristor can operate with multilevel storage capacity and can also exhibit volatile (diffusive)/nonvolatile (drift) switching to provide various platforms, such as neuromorphic applications as neurons or synapses, with the same materials in the device.^45,46^ Therefore, for memristors with this type of switching, the physical properties of the CFs and which species formed the CFs are very important. In HP-based memristors driven in such a filamentary-type switching, the elements that make up the CFs are typically reported in two types: One is metallic CFs related to electrochemical metallization (ECM) due to the redox reaction of the top electrodes, and the other is vacancies/anions related to the valence change mechanism (VCM) due to the defect migration in the HP layer. (The detailed mechanism will be discussed in a further section.) The components of the CFs depend on the device structure, such as top/bottom electrodes, the thickness of the active switching layer, etc.^40^ This is because it depends on which species has a low energy barrier to movement and moves more easily to form the CFs, related to the activation energy of the ion migration species.^21,23^ Especially, defects caused by halide anions have been reported to have a low energy barrier to ion migration, making them more likely to form CFs than defects in other species, such as V_A_ and V_B_ in ABX_3_ HPs. As such, HP-based memristors are most reported to form the CFs in the switching medium because they have low activation energy for ionic/electronic migration, such as halide migration throughout the HP-based switching medium.

On the other hand, memristive devices driven by interface-type switching are also reported, where the vacancy movement process, the trapping/detrapping process of charge carriers, or the transition of doped carriers occurs at the interface depending on the type of electrode, the thickness dependence of the HP layer, the insertion of other layers in the device, etc.^40−42,47^ In a memristor with interface-type switching, the resistance changes occur at the interface between the electrode and the switching active layer or at the hybrid interface between two medium layers, caused by modification of the barrier height at the interface between the metal electrode and semiconductor layer (Figure 2A).^40−42,47−49^ Since the barrier height and reactivity between two layers play an important role in the interface-type switching devices, the RS memory effect can be regulated by controlling the charge transfer at the contact interface with switching active HP layer. Solanki et al. analyzed the *I*–*V* hysteresis of the interface-type switching memristor with impedance spectroscopy (IS) by tuning the characteristic structural flexibility of HPs to low dimensions according to the composition and appropriate selection of external interfaces in the device, such as electrodes between Ag (more active) and Au (less active).^48^ They utilized different structural HPs with the chemical formula of (PEA)_2_(MA)_*n*−1_Pb_*n*_I_3*n*+1_ (PEA = phenylethylammonium; MA= methylammonium; *n̅* = 1, 3, 5, 7, ∞) for the memristive device of ITO/PEDOT:PSS/HP/PCBM/Ag. Depending on the PEA layer, which is a nonconductive organic spacer, 2D layered low-dimensional (*n̅* = 1), quasi-2D (mixed) Ruddlesden–Popper (RP) HP, and 3D HP (*n̅* = ∞) structures were formed (according to the value of *n̅*, where the PEA layer affected the OFF state (HRS) with relatively few mobile ions. As such, they investigated the physical and chemical reaction of ions moving between the HP and the contact interface by using the characteristic structural flexibility of HP and the contact interfacial reactivity with a combination of *I*–*V* characteristics and IS analysis and proposed a method to improve memristive properties by adjusting the HP composition/dimensions and interfacial contact materials. As such, the change in conductance occurs throughout the interface between the electrode and active layer or the hybrid interface between two other layers, so the selection of the type or area of the electrode and the selection of the appropriate switching material play important roles in this type of switching device. Also, the resistance value is relatively affected by the area of the electrode in the interface-type switching; sometimes these two types can be simply distinguished by analyzing the tendency of the current level according to the size of the electrode in the memristor *I*–*V* analysis.

For example, Choi et al. reported solution processed CH_3_NH_3_PbI_3_-based memristor, showing high ON/OFF ratio over 10^6^ with ultralow operation voltage below 0.15 V.^21^ To investigate the current conduction mechanism and switching types of the Ag/CH_3_NH_3_PbI_3_/Pt memristor, the *I*–*V* characteristics were confirmed after the top electrode was changed to Ni and Au or the size of the Ag electrode was increased by 64 times. The electrodes replaced by Ni and Au produced higher driving voltages, and the changed electrode’s size did not make a notable change in the resistance value. By colligating these results, it was confirmed that its abrupt RS behavior occurred through the localized Ag CF type switching, which was also confirmed indirectly by comparing the current map on the thickness of the HP-film with grain boundaries etc. in conducting AFM (c-AFM). Guan et al. showed interface-dominated RS devices using different thickness of HPs in the same MAPbBr_3_-based memristor and also showed different types of filamentary switching curves according to different compliance currents in the MAPbI_3_-based memristor that replaced Br with I.^41^ As shown in Figure 2B, Han et al. also implemented the memristor showing the distinct mechanisms of filamentary-type and interface-type switching through the electrode engineering of the device design in the same all-inorganic tin iodide HP-based memristor.^40^ In this way, even the memristor using the same HP can be driven by different switching mechanisms by a number of complex factors and shows a different switching type, whether it is filamentary or interface type switching. As shown in Figure 2C, Guan et al. investigated the dependence of the switching mechanisms by varying the thickness and composition of the active switching HP layer in the MAPbBr_3_-based memristors. They explained by exhibiting different aspects of *I*–*V* characteristics that are driven by interface-type RS behavior in the memristor of a thicker HP layer up to 1 μm and by filamentary-type RS behavior in the memristor of a thinner film of 350 nm.^41^ Also, Chen et al. fabricated the HP-based artificial synaptic device of Ag/MoO_3_/CsPbI_2_Br/MoO_3_/ITO to exhibit the synaptic characteristics such as PPF, STP, and LTP (Figure 2D).^42^ The memristive behavior of the synaptic device was shown to be caused by the formation/annihilation of the AgI composite layer by diffusion/distribution of Ag cations and halide anions (I^–^) by an external electric field at the hybrid interface between MoO_3_ and the HP layer. They confirmed the resistive switching of HRS and LRS at the interface between two layers (MoO_3_/HP) through energy-dispersive X-ray spectroscopy (EDS) and TEM analyses and further transformed the Ag top electrode into a Au electrode for their hypothesis verification and proved it through *I*–*V* analysis depending on the presence/absence of the AgI layer at the interface between MoO_3_ and the HP layer.

As such, the HP-based memristive devices can affect the switching types of every layer of the device’s overall structure, including which electrodes are used, which layers are stacked, and what the structure and characteristics of the switching active layer (here, mainly HP layer) are.

### 2.2 Switching Mechanisms on HP-Based Memristors

**Figure 3:**
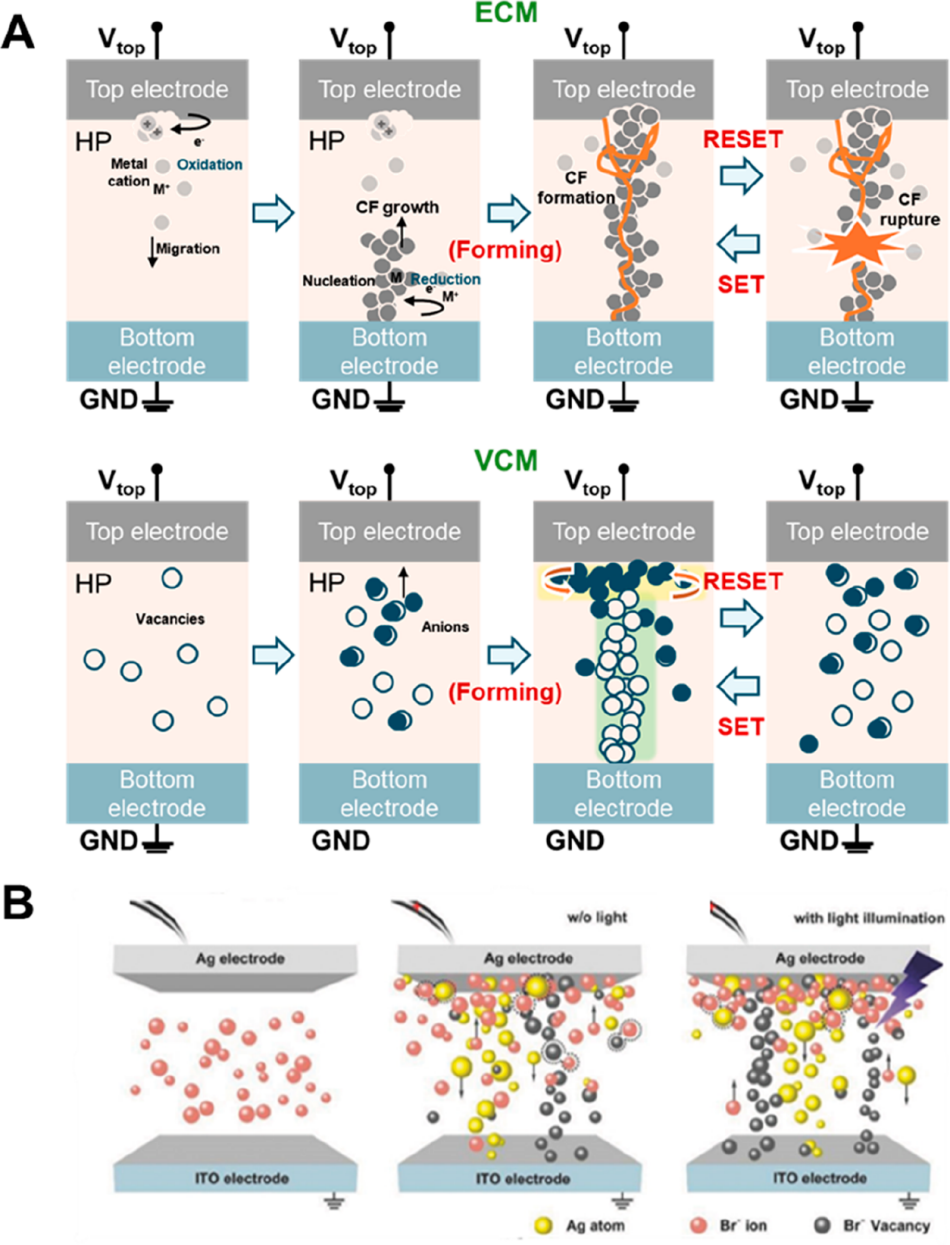
(A) Schematic electrochemical metallization (ECM, cation-type) and valence change mechanism (VCM, anion-type). (B) Schematic process with synergetic cationic–anionic switching mechanisms. Reproduced from Wang, Y.; Lv, Z.; Liao, Q.; Shan, H.; Chen, J.; Zhou, Y.; Zhou, L.; Chen, X.; Roy, V. A. L.; Wang, Z.; Xu, Z.; Zeng, Y.-J.; Han, S.-T. Synergies of Electrochemical Metallization and Valence Change in All-Inorganic Perovskite Quantum Dots for Resistive Switching. *Adv*. *Mater*. **2018**, 30 (28), 1800327.^50^ Copyright 2018 John Wiley and Sons.

There are largely two typical types of switching mechanisms on HP-based memristive devices, cationic migration (typical electrochemical metallization, ECM) and anionic migration (typical valence change mechanism, VCM), as illustrated in Figure 3A. The criteria for dividing ECM and VCM mechanisms depend on the type of predominant mobile species that make up the conductive pathway. The electrochemical metallization (ECM) mechanism is driven through the oxidation reaction of metal electrodes in the active layer when an electrochemically active metal such as Ag or Cu is used as the top electrode with an inert counter electrode (Au, Pt, etc.) in the MIM structure (upper part of Figure 3A). When an external electrical stimulus is applied to the device, the electrochemically active metal is oxidized (M → M^+^ + e^–^) at the first step, and the metal cation moves toward the counter electrode and is reduced (M^+^ + e^–^ → M) as the nucleation of metal ions at the second step, growing the localized conductive pathway. As the channel grows, the conductive bridge finally is formed between two electrodes with metallic Ohmic conduction, causing the resistance change of the memristor and turning it to the ON state. When the electrical bias is given in the opposite direction, these formed metallic CFs are dissolved mainly through the Joule heating effect in the HP-based memristors, turning the device to the OFF state. In this mechanism, the electroforming step that requires higher electric fields at the first switching step for a stable repetitive switching process often appears. That process can hinder practical applications, so electroforming-free memristors have been reported by controlling these redox reactions through sophisticated modulation of the device materials and devices. For example, Im et al. successfully produced an electroforming-free switching device through Ag doping in MAPbI_3_-based memristive devices. Due to prelocated Ag atoms, higher concentrations of Ag are included in a Ag filament formation process and the Ag flux slows down to make the filament larger and prolong the lifetime, resulting in the electroforming-free ECM-based memristor.^51^

In the valence change mechanism (VCM) illustrated in the bottom part of Figure 3A, anionic migration mainly engages in the conduction mechanism, where anions or anion vacancies such as halide anion/vacancies within the active switching medium form the conducting channels, causing the change in the valence state of the materials. With an ionic nature, HPs (composed of cation of A-site and B-site and anion of X-site) form various vacancies easily and quickly inside due to their species’ low defect formation energy (activation energy), creating the resistance change within the switching layer. In particular, X-site halide anions are reported to have a much lower migration activation energy barrier than the cations of the other two sites.^52−54^ It often changes the conductivity within the HP layer through synergetic ion migration between the vacancies of A-site or B-site and halide ions for memory and neuromorphic devices.^55,56^ To prove these properties on mechanism analysis, the calculation involving halide vacancies and ion migrations in the HP-layer with first-principles based density functional theory (DFT) has also been reported. Through DFT, the ionic migration barriers for each halide anion were clarified, making it possible to calculate, prove the abundant mobile ion species, and screen the number of possible candidate memristors.^23,32,57−59^

Since the mobile ionic migration or defect migration also tends to take place along the grain boundaries formed in HP-based polycrystalline films, defect engineering is one of the ways to induce the ionic migration for the operation of VCM-based memristors and define the mechanism principles, which can be confirmed by using various microscopies or spectroscopies.^60−63^ (Various analysis methods will be described in the following section.) Zhang et al. modulated the grain sizes and boundaries in the CH_3_NH_3_PbI_3_-based optoelectronic memristor through a polyacrylonitrile (PAN) passivation method, compared the RS parameters, and explained the switching mechanism.^63^ In their CH_3_NH_3_PbI_3_-based memristors driven by iodide migration (VCM), the reduced ion mobility in devices with fewer grain boundaries reduced the randomness of CFs and made RS performance stable because the activation energy of iodide migration at grain boundaries was lower than that at the grain interior. HP also has various crystal structures and flexible dimensionality, from the typical 3D ABX_3_ HP to low-dimensional HP (0D, 1D, 2D, quasi-2D) depending on the composition. It makes different film morphologies, defect formation energies, and bandgaps depending on the type and ratio of composition at each site, which can also change device driving characteristics.^48,64−67^ Jeong et al. fabricated 3D FAPbBr_3_- and quasi-2D (RNH_3_)_2_(FA)_1_Pb_2_Br_7_-based VCM-driven memristors (FA = CH(NH_2_)_2_PbI_3_) stacked in the structure of Au/HP/ITO.^65^ They investigated the memristive properties and operating mechanisms, where the quasi-2D HP-based device showed nonvolatile write-once read-many (WORM) memory effects but 3D HP-based devices showed only hysteresis behavior. Nanoscale morphology analysis and current mapping were utilized for the analysis of HP grain boundaries. The organic ligands (here, oleylammonium) used to form quasi-2D HPs in 3D HPs served to passivate and protect the major channel for high current, which caused quasi-2D HPs to show relatively fewer Br vacancies/defects across the GB and improved RS memory characteristics.

Unlike the ECM mechanism, which operates only in the presence of metal species with high electrochemical activity, the VCM can occur in both cases, where the top electrode is an electrochemically active metal or inert metal. When the top electrode is composed of an electrochemically active metal, the memristive device creates a competitive conductive flow depending on the external stimuli, such as driving voltage or thickness of the switching active layer that affects which of both species (metallic cation, vacancies of internal HP) forms the predominant conductive pathway.^64,65^ For example, in an MAPbI_3_-based memristor, the mechanisms and RS characteristics were investigated according to the thickness control of the perovskite layer, the device driving voltage, and the change of the top electrode.^69^ In this research, it was described that ECM driving (Ag migration) was performed in the case of having a thin HP layer or operating an electric field large enough to promote Ag movement in the thick perovskite layer. Similarly, studies on a method of controlling ion mobility by varying thickness control and driving method for the desired driving mechanisms (ECM/VCM)/types of targets are also being actively pursued. (More detailed methods and efforts are in section 2.5.)

ECM-based memristors are mainly driven by the filamentary switching type, while VCM-based memristors can be driven by the localized CF formation/rupture made of anionic vacancies across the switching layer between two top-bottom electrodes (vertically shaded area in the third step) or interface-type switching (horizontally shaded area in the third step) by vacancies that are distributed across the interface between the top electrode and HP-based active layer in the bottom part (VCM) of Figure 3A. It is possible to make a simple test of whether the device exhibits ECM or VCM by using the top electrode as an electrochemically inactive electrode and then analyzing the presence or absence of resistive switching properties or operating *I*–*V* characteristics. This is because the relationship between the metal electrode and switching active layer (here, HP layer) in the MIM structure has a predominant impact on ECM- and VCM-driven devices.^48^

However, HP-based memristors always have intrinsic anionic/point defects and complex characteristics, so it is an ideal fragment scenario that the mechanism is always completely distinguished by switching with metal cations or vacancies. In this regard, it also has been suggested that the conductive pathway can be synergistically formed in the form of dual filaments (part metallic and part vacancy CF) or in the form of AgX composition of combined oxidized Ag^+^ reservoir of highly reactive metal (Ag) and halide anions/vacancies (X^–^) (Figure 3B).^50,69−72^ In other words, the HP-based memristor could also be operated by a synergetic effect between ECM and VCM, where V_X_^+^ halide vacancies of well-known n-type dopant made from halide anion (X^–^) and electrochemically active metal cation (M^+^) such as Ag^+^ produce the RS progress by causing the energy band shift and the reaction in the active HP-based switching layer. Of course, even in all these cases, there are specific species and mechanisms that contribute more heavily to driving the conductive pathway.

### 2.3 Abrupt Switching and Gradual Switching Operation

**Figure 4:**
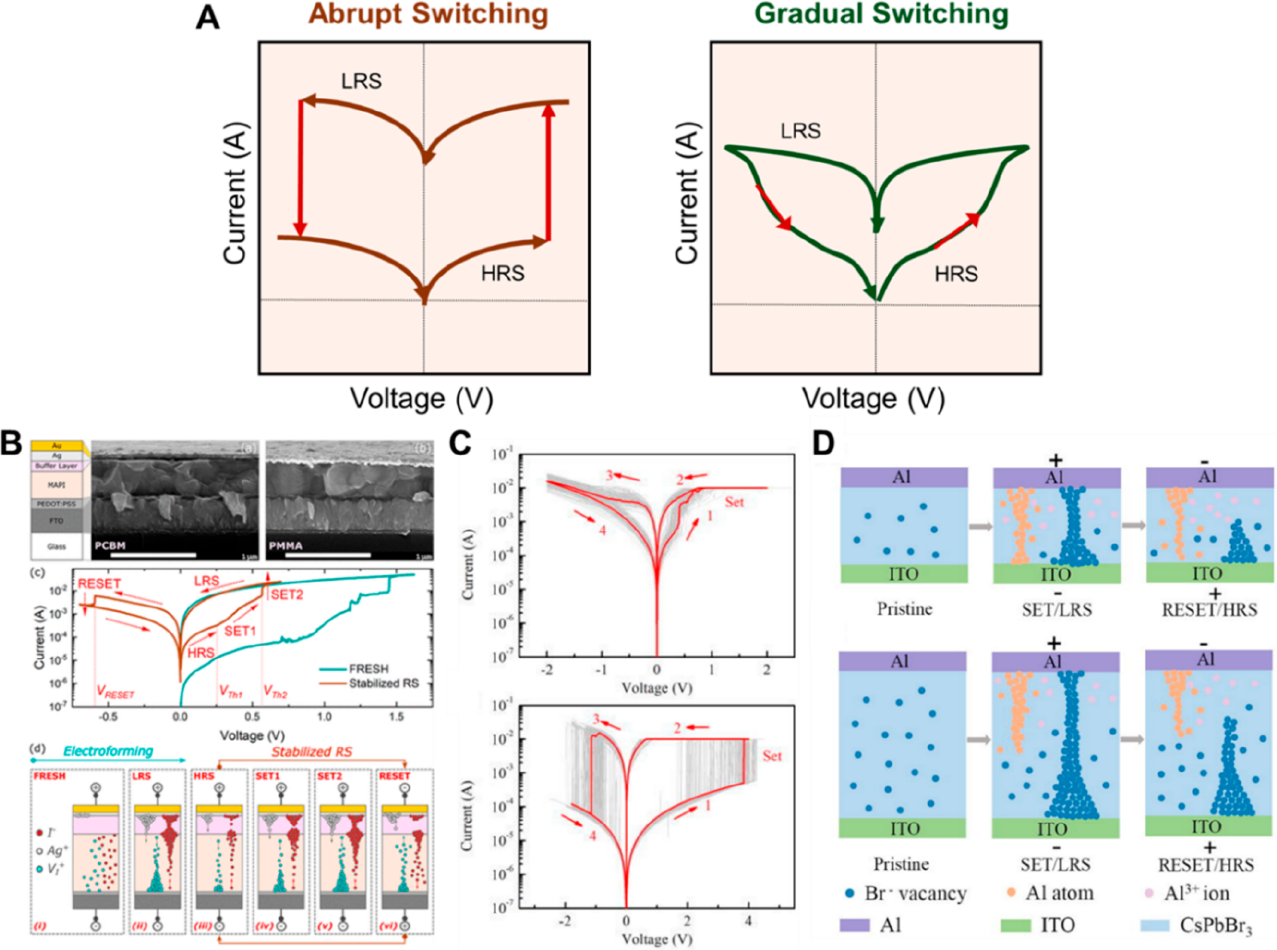
(A) *I*–*V* schemes of bipolar memristor divided to abrupt switching (left) and gradual switching (right). (B) *I*–*V* characteristics and schematic switching process showing the two-step switching sweeps of abrupt switching and gradual switching processes through buffer layer insertion. Reproduced from Gonzales, C.; Guerrero, A. Mechanistic and Kinetic Analysis of Perovskite Memristors with Buffer Layers: The Case of a Two-Step Set Process. *J Phys*. *Chem*. *Lett*. **2023**, 14, 1395.^73^ Copyright 2023 American Chemical Society. (C) *I*–*V* curves of gradual (analog) and abrupt (digital) switching and (D) diagram showing the main operating mechanism according to the film thickness. Digital abrupt RS behavior (thick film, applied for data storage field) and analog gradual RS behavior (thin film, applied for artificial synapse) of CsPbBr_3_-based memristive devices according to the thickness of the switching film. Reproduced from Zhu, Y.; Chen, M.; Lu, H.; Mi, P.; Luo, D.; Wang, Y.; Liu, Y.; Xiong, R.; Wang, H. The Filaments Control for Tunning Digital Resistive Switching in Data Storage Application and Analog Behavior as an Artificial Synapse with CsPbBr_3_–Based Memristor. *Appl*. *Phys*. *Lett*. **2024**, 124, 063504.^74^ Copyright 2024 AIP Publishing.

In relation to these switching mechanisms, there are two types of characteristic behaviors seen in *I*–*V* sweep modes in the memristors: the abrupt switching process and gradual switching process (Figure 4A).^75−77^ Depending on these two switching transition types, it is important for applications to obtain the desired sweep from *I*–*V* characteristics because gradual switching is relatively beneficial to neuromorphic analog synaptic plasticity and abrupt switching is beneficial to digital high-capacity memory storage systems. In abrupt switching operation, the resistance changes between HRS and LRS occur rapidly and suddenly after the application of an external electric field, while in the gradual switching operation, they occur gradually with multiconductance states. Generally, the abrupt type switching mode shows very fast switching speed and high ON/OFF ratio, enabling the memristors to be applied to ultrafast and high-density digital memory systems with rapid variations.^39^ The gradual type switching mode usually shows a relatively controllable transition, which is more beneficial to be applied to emulate artificial synapses of neuromorphic computing systems with analog switching sweeps.^76^ Even if the same HP is used for the memristor, the form of the conduction transition varies depending on the operation mechanism of the memristor, which means the application fields may also vary accordingly.

To target among these two types of switching dynamics, the modulation of switching the active HP layer and the device structure is especially important because it affects carrier transport/ion or defect migration dynamics in memristive devices. In all-inorganic lead-free HP-based memristors of the same composition of HP, Han et al. changed only the top electrode from silver electrodes (Ag) to gold electrodes (Au) in the device, and the different types of switching transitions were demonstrated. The filamentary-type abrupt switching transition was found under Ag electrodes (Ag^+^ migration), and the interface-type gradual switching transition was found under Au electrodes (Sn^2+^ vacancy migration) (Figure 2B).^40^ In this regard, Gonzales et al. exhibited the transition switching dynamics with the dynamic IS in 2D RP HP-based memristors. The transition switching speed for the Ag and Spiro/Ag ReRAM devices was monitored by chronoamperometry measurements, showing the gradual transition of the Ag ReRAM and the abrupt transition of the Spiro/Ag ReRAM. They explained the view of kinetic phenomenon, which is varied by ion migration and redistribution occurring at HP and metal contact interfaces.^78^ As a follow-up study, they inserted buffer layers (6,6-phenyl C61 butyric acid methyl ester, PCBM, or poly(methyl methacrylate), PMMA) between the top Ag electrode and MAPbI_3_ HP without any doping and analyzed the switching mechanism mechanically and kinetically (Figure 4B).^73^ Unlike the memristor without a buffer layer, the two memristors with buffer layers showed two-step switching behaviors: abrupt and gradual switching. It was explained that the drift/diffusive switching response of the two-type switching process is the result of charge accumulation and the interfacial reaction by controlling the dynamics of ion migration to the buffer layer and appropriately adjusting the reactivity of ions moving with Ag, and an inspection of the switching mechanism was also provided using a dynamic model in each process. Lee et al. inserted an ALD-deposited SnO_2_ layer between δ-FAPbI_3_ HP layer and ITO bottom electrode, and an artificial synapse was implemented by changing the *I*–*V* characteristics into a gradual switching behavior.^79^ They also created a heterojunction structure by inserting additional layers into the memristor device and changed the physical switching behavior by controlling ion migration at the interface with the HP layer. Ganaie et al. showed abrupt digital switching and gradient analog switching by converting only anions in benzyl ammonium (BzA)-based Ruddlesden–Popper low-dimensional HPs, where it is interpreted that the gradual movement of iodide ion resulted in analog switching and the faster ion migration of bromide ion with lower activation energy produced rapidly changing CFs of halide vacancies.^80^ As presented in Figure 4C,D, Zhu et al. implemented the abrupt–gradual switching tunability by changing the thickness of the CsPbBr_3_-based HP layer under an adaptive electrode (here, Al). In a thin film, bromide vacancies and aluminum atoms can form CFs together, but nonsynchronic CFs are formed and destroyed by different migration energies of the two species, causing an analog switching phenomenon. On the other hand, in the memristor with a thick HP layer, the conducting channel between two electrodes was formed mainly by bromide vacancies due to the high formation free energy of aluminum, resulting in a more rapid digital RS memristor.^74^

According to these reported studies, abrupt and gradual transitions may occur on the same device and also occur due to the changes in the conductive pathway caused by stacking type/materials/thickness of the memristive devices. Especially, it can be seen that the interface contact and ionic species in the switching active layer (here, HP layer) of the memristive devices has a significant impact on the switching transition physics.^81−83^ Various efforts, such as making a heterojunction layered device or physical modulation of the HP layer, have recently been made to analyze switching behaviors and identify gradual/abrupt conductance changes in connection with mechanisms to implement appropriate applications of neuromorphic devices and memristors, but some difficulties remain in modulating these two types of switching dynamics without clear mechanism analysis. There are still not many prior studies focusing on the analysis of these dynamics, so it is clear that more research should be done on the physics and transition time response. We summarize the recently reported HP-based memristive devices’ performance and switching mechanisms in Tables 1 and 2.

**Table 1:** Halide Perovskite-Based Memristors (Memory Devices)

Device Structures	Operating Voltages (V_set_/V_reset_) [V]	ON/OFF ratio	Endurance [cycles]	Switching Type	Switching Mechanism	Switching Operation	Ref.
EGaIn/MA_0.85_Cs_0.15_PbI_3_/PEDOT:PSS/ITO	+0.54/–0.66	10^5^	2000	Filamentary	VCM (Halide vacancy)	Abrupt	(12)
Au/MAPbI_3-x_Cl_*x*_/FTO	+0.8/–0.6	10^4^	100	Interface	trap-filled SCLC	Gradual	(19)
Ag/CH_3_NH_3_PbI_3_/Pt	+0.15/–0.15	10^6^	350	Filamentary	ECM (Ag migration)	Abrupt	(21)
Au/Cs_3_Sb_2_I_9_/ITO	>+1.0/–1.0	10^2^	500	Filamentary	VCM (Halide vacancy)	Abrupt	(23)
Ag/BA_2_CsAgBiBr_7_/Pt/Ti/SiO_2_/Si	+0.13/–0.2	10^7^	1000	Filamentary	ECM (Ag migration)	Abrupt	(30)
Ag/BA_2_MA_n-1_PbnI_3n+1_/Pt	+0.5/–0.6	10^7^	250	Filamentary	ECM (Ag migration)	Abrupt	(31)
Ag/FAPbI_3_/Pt	+0.22/–0.15	10^5^	1200	Filamentary	VCM (Halide vacancy)	Abrupt	(32)
Au/Cs_3_Bi_2_I_9_/Pt/Ti/SiO_2_/Si	+0.1/–0.27	10^8^	400	Filamentary	VCM (Halide vacancy)	Abrupt	(34)
Au/Rb_3_Bi_2_I_9_/Pt/Ti/SiO_2_/Si	+0.09/–0.24	10^7^	200	Filamentary	VCM (Halide vacancy)	Abrupt	(34)
Ag/PMMA/(BzA)_2_CuBr_4_/Pt	+0.2/–0.3	10^8^	2000	Filamentary	ECM (Ag migration)	Abrupt	(36)
Al/CsPbBr_3_/PEDOT:PSS/ITO/PET	–0.6 /+1.7	10^2^	50	Filamentary	ECM (Al migration)/VCM (Halide vacancy)	Gradual/Abrupt	(37)
Au/CsPb(Br_0.93_I_0.07_)_3_ QDs/ITO	+0.92/–3.01	10^3^	100	Filamentary	VCM (Halide vacancy)	Gradual/Abrupt	(38)
Ag/PMMA/CsSnI_3_/Pt/Ti/SiO_2_/Si	+0.13/–0.08	7 × 10^3^	600	Filamentary	ECM (Ag migration)	Abrupt	(40)
Au/PMMA/CsSnI_3_/Pt/Ti/SiO_2_/Si	+0.5/–1.5	10^3^	120	Interface	VCM (Sn vacancy)	Gradual	(40)
Au/MAPbBr_3_/ITO	+1/–1	10^3^	1000	Interface	trap-filled SCLC/MA vacancy	Gradual	(41)
Ag/Cs_3_Bi_2_Br_9_/ITO	+0.44/–1.5	10^7^	2000	Filamentary	VCM (Halide vacancy)	Gradual	(44)
Ag/MoO_*x*_/RbPbI_3_/ITO	+0.35/–1.25	10^2^	2000	Filamentary	ECM (Ag migration)	Gradual	(45)
ITO/PEDOT:PSS/PEA_2_MA_4_Pb_5_I_16_/PCBM/Ag	+0.5/–1.0	10^4^	500	Interface	VCM (Halide vacancy)/AgI formation	Gradual	(48)
ITO/MAPbI_3-x_Cl_*x*_/BAI/Al	+0.79/–0.77	10^3^	300	Interface	trap-filled SCLC	Abrupt	(49)
Ag/PMMA/CsPbBr_3_ QDs/PMMA/ITO/PET	+1.1/–1.7 (light-assisted)	10^5^	5000	Filamentary	ECM + VCM filament	Abrupt	(50)
Ag/MAPbI_3_:Ag/Au	+0.34/–0.32	10^5^	3000	Filamentary	ECM (Ag migration)	Abrupt	(51)
Au/CH_3_NH_3_PbI_3_:PAN/FTO	>+0.5/–0.5	10^2^ ∼ 10^5^	200	Filamentary	VCM (Halide vacancy)	Abrupt	(63)
Ag/(PEA)_2_PbI_4_/ITO	+0.15/–0.06	10^4^	1000	Filamentary	ECM (Ag migration)	Abrupt	(67)
Ag/MAPbI_3_/FTO	>+1.0/–1.0	10^6^	1000	Filamentary	ECM (Ag migration)/VCM (Halide vacancy)	Abrupt	(68)
Ag/PMMA/MAPbI_3_/FTO	–0.1/+0.9	10^6^	2000	Filamentary	VCM (Halide vacancy)/AgI formation	Abrupt	(69)
Ag/PCBM/BA_2_MA_3_Pb_4_I_13_/NiO_*x*_/ITO	+0.4	10^6^	400	Filamentary	VCM (Halide vacancy)/AgI formation	Abrupt	(70)
Ag/CsAgInCl_6_/ITO	+1.2/–1.2	10^3^	1000	Filamentary	ECM + VCM filament	Abrupt	(71)
Au/CsAgInCl_6_/ITO	+1.2/–1.2	10^4^	1500	Filamentary	VCM (Halide vacancy)	Abrupt	(71)
Au/Ag/Buffer Layer/MAPbI_3_/PEDOT:PSS/FTO	+0.60/–0.59	39.3	12000	Filamentary	VCM (Halide vacancy)/AgI formation	Gradual	(73)
Al/CsPbBr_3_/ITO	+0.3/–0.37	10^3^	300	Filamentary	ECM (Ag migration)/VCM (Halide vacancy)	Gradual/Abrupt	(74)
Al/(BzA)_2_PbX_4_/FTO	+1.04/–1.33	>10^2^	500	Filamentary	VCM (Halide vacancy)	Gradual/Abrupt	(80)
Au/CH_3_NH_3_PbI_3_/TiO_2_/FTO	+0.85/–1.4	10^3^	350	Interface	charge trapping/VCM (Halide vacancy)	Gradual	(82)
Ag/PMMA@CsPbI_3_/FTO	+0.4/–0.4	10^2^	500	Filamentary	ECM (Ag migration)	Gradual	(84)
Ag/MoO_3_/CH_3_NH_3_PbI_3_/ITO	+0.27/–0.57	>10^2^	1100	Filamentary	ECM (Ag migration)	Gradual/Abrupt	(85)

**Table 2:** Halide Perovskites-Based Memristors (Neuromorphic Devices)

Device Structures	Energy Consumption	Operating Mechanisms	Ref.
Al/MAPbBr_3_/Al/BCCP/ITO	20 fJ/spike	Ion migration (Br^–^) (VCM)	(20)
Al/PEA_2_PbBr_4_/BCCP/ITO	0.7 fJ/spike	Ion migration (Br^–^) (VCM)	(22)
Au/BCP/C_60_/MA_0.825_FA_0.275_Pb_0.75_Sn_0.25_I_3_/PEDOT:PSS/ITO	0.6 nJ/spike	Ion migration (I^–^) (VCM)	(29)
Ag/PMMA/MA_3_Sb_2_Br_9_/ITO	117.9 fJ/μm^2^	Ion migration (Br^–^) (VCM)	(35)
Ag/MoO_3_/CsPbI_2_Br/MoO_3_/ITO	2 nJ/mm^2^	Ion migration (I^–^) and metal atoms (Ag+) migration (ECM + VCM)	(42)
Ag/MoO_*x*_/RbPbI_3_/ITO	15.69 fJ/μm^2^	Ion migration (I^–^) (VCM)	(45)
Ag/CsPbBr_3_ NCs/pTPD/PEDOT:PSS/ITO	–	Ion migration (Br^–^) and metal atoms migration (ECM + VCM)	(46)
Au/PMMA/PEA_2_MA_4_Pb_5_I_16_/ITO	–	Ion migration (I^–^) (VCM)	(60)
Al/PMMA/Cs_3_Sb_2_I_9_/ITO	10 fJ/μm^2^	Ion migration (I^–^) and Sb vacancy (VCM)	(66)
Au/Cs_2_AgInCl_6_/ITO	–	Ion migration (Cl^–^) (VCM)	(71)
Ag/Cs_2_AgInCl_6_/ITO	–	Ion migration (Cl^–^) and metal atoms (Ag+) migration (ECM + VCM)	(71)
Al/CsPbBr_3/_ITO	–	Ion migration (Br^–^) and metal atoms migration (ECM + VCM)	(71)
Ag/δ-FAPbI_3_/ALD-SnO_2_/ITO	–	Ion migration (I^–^) (VCM)	(78)
Al/(BzA)_2_PbI_4_/FTO	–	Ion migration (I^–^) (VCM)	(80)
Au/MAPbBr_3_ SC/Au	0.54 aJ/μm^2^	Ion migration (Br^–^) (VCM)	(94)
Au/MAPbBr_3_/Au (lateral)	14.3 fJ/spike	Ion migration (Br^–^) (VCM)	(54)
Ca/Al/Bphen/MAPbBr_3_/PEDOT:PSS/ITO	34 nJ/mm^2^	Ion migration (Br^–^) and MA vacancies (VCM)	(55)
Ca/Al/Bphen/FAPbBr_3_/PEDOT:PSS/ITO	23 nJ/mm^2^	Ion migration (Br^–^) and FA vacancies (VCM)	(55)
Ca/Al/Bphen/CsPbBr_3_/PEDOT:PSS/ITO	153 nJ/mm^2^	Ion migration (Br^–^) and Cs vacancies (VCM)	(55)
Au/MAPbI_3_/PEDOT:PSS/ITO	55 fJ/(100 nm)^2^	Ion migration (I^–^) and MA vacancies (VCM)	(56)
Au/FABi_3_I_10_-FA_3_Bi_2_I_9_ mixed phase/PEDOT:PSS/ITO	61.08 aJ/spike	Ion migration (I^–^) (VCM)	(57)
Ag/PMMA/Cs_2_AgBiBr_6_/ITO	188.6 pJ/spike	Ion migration (Br^–^) and metal atoms (Ag+) migration (ECM + VCM)	(72)
Au/Cs_0.22_FA_0.78_Pb(I_0.85_Br_0.15_) _3_/ITO/PEN	0.1 fJ/spike	Ion migration (Br^–^) (VCM)	(100)
Au/PEA_2_PbBr_4_/Graphene	400 fJ /μm^2^	Ion migration (Br^–^) (VCM)	(101)
Al/MAPbClBr_2_/Si	5.8 pJ/spike	Ion migration (Cl^–^) (VCM)	(102)
Au/Cs_3_Cu_2_I_5_/PEDOT:PSS/ITO	10.48 aJ/μm^2^	Ion migration (I^–^) (VCM)	(103)

### 2.4 Electrical and Physical Analysis for Understanding the Switching Mechanisms

**Figure 5:**
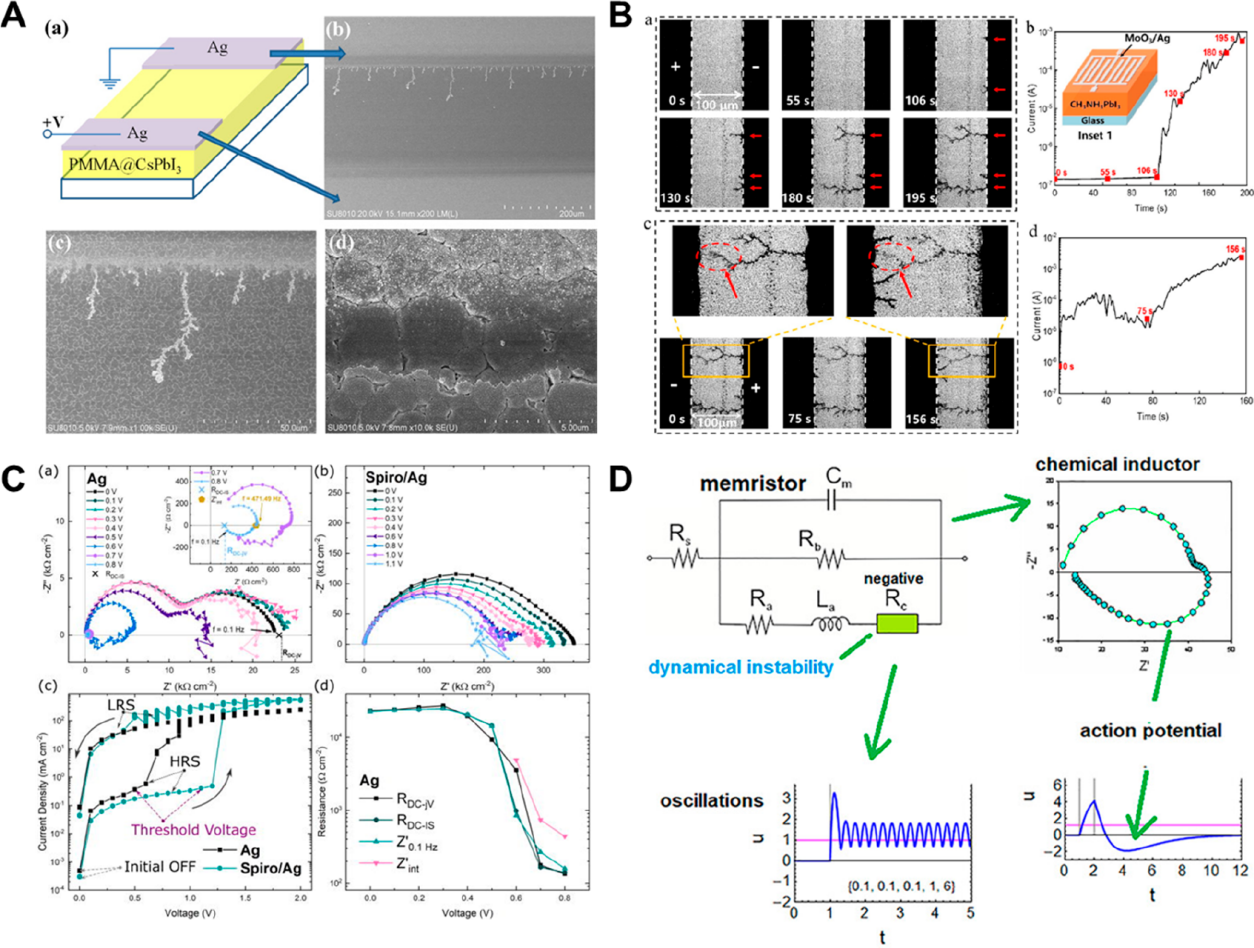
(A) Formation process of Ag filaments during applied voltage shown in top-view SEM images with schematic device structure. Reproduced from Xu, J.; Wu, Y.; Li, Z.; Liu, X.; Cao, G.; Yao, J. Resistive Switching in Nonperovskite-Phase CsPbI_3_ Film-Based Memory Devices. *ACS Appl*. *Mater*. *Interfaces***2020**, 12, 9409.^84^ Copyright 2020 American Chemical Society. (B) PL imaging microscopy tracking CF formation processes with time under applied voltage on the lateral HP-based memristor. Reproduced from Luo, H.; Lu, L.; Zhang, J.; Yun, Y.; Jiang, S.; Tian, Y.; Guo, Z.; Zhao, S.; Wei, W.; Li, W.; Hu, B.; Wang, R.; Li, S.; Chen, M.; Li, C. In Situ Unveiling of the Resistive Switching Mechanism of Halide Perovskite-Based Memristors. *J Phys*. *Chem*. *Lett*. **2024**, 15 (9), 2453.^85^ Copyright 2024 American Chemical Society. (C) HP-based memristor which contains enormous inverted hysteresis in IS spectra. Reproduced from Gonzales, C.; Guerrero, A.; Bisquert, J. Spectral Properties of the Dynamic State Transition in Metal Halide Perovskite-Based Memristor Exhibiting Negative Capacitance. *Appl*. *Phys*. *Lett*. **2021**, 118, 073501.^78^ Copyright 2021 AIP Publishing. (D) Physical dynamic model of HP-based memristor for artificial neurons and electrical response over time. Reproduced from Bisquert, J.; Guerrero, A. Dynamic Instability and Time Domain Response of a Model Halide Perovskite Memristor for Artificial Neurons. *J*. *Phys*. *Chem*. *Lett*. **2022**, 13, 3789.^86^ Copyright 2022 American Chemical Society.

Various efforts have been made to analyze these complicated driving mechanism principles of HP-based memristors and neuromorphic devices in various aspects (Figure 5). With a greater focus on high performance and application, the simplest and most common switching mechanism investigation has been analyzed indirectly by fitting the slopes and values in the logarithm *I*–*V* curves and measuring the *I*–*V* tendency according to temperature changes, the changes of the electrodes, etc. The conduction flow mechanisms in HRS and LRS also have been investigated by substituting various equations presented as existing memristor conduction mechanisms as a comprehensive analysis in the *I*–*V* curve obtained in HP-based memristors (e.g., Ohmic conduction, Schottky conduction, SCLC, hopping conduction, trap-assisted tunneling, Poole–Frenkel emission, etc.).^87,88^

However, to substantiate the results obtained from fitted values and experimental data in more depth, several efforts have been added to analyze the mechanisms with direct and indirect visibility through microscopic and spectroscopic methods such as atomic force microscopy (AFM), *in situ* photoluminescence (PL) imaging, transmission electron microscopy (TEM), scanning electron microscopy (SEM), analysis of valence change through the X-ray photoelectron spectroscopy (XPS), energy dispersive spectroscopy (EDS) mapping, electron energy loss spectroscopy (EELS) and secondary ion mass spectroscopy (SIMS), glow discharge optical emission spectroscopy (GD-OES), impedance spectroscopy (IS), etc. In the early stages of HP-based memristor research, Choi et al. reported a solution-processed CH_3_NH_3_PbI_3_-based memristor, showing high ON/OFF ratio over 10^6^ with ultralow operation voltage below 0.15 V.^21^ To see the switching mechanism of the Ag/CH_3_NH_3_PbI_3_/Pt memristor, the *I*–*V* characteristics were confirmed after the top electrode was changed into Ni and Au or the size of the Ag electrode was increased by 64 times. By colligating these results, it was confirmed that the abrupt RS behavior occurred through the localized CF of the electrochemically active Ag electrode, which was also confirmed indirectly by comparing the current map on the thickness of the HP film with grain boundaries, etc. in conducting AFM (c-AFM). Han et al. reported lead-free all-inorganic CsSnI_3_-based memristors with Ag and Au top electrode and compared the two distinct switching mechanisms through logarithmic *I*–*V* plots and electrode/temperature dependence.^40^ They also examine the switching mechanisms in CsSnI_3_-memristors through time-of-flight (ToF) SIMS. The memristor with Ag electrode showed the fluctuated Ag distribution in the HP active layer after operation, which means ECM filamentary switching, while the memristor with Au electrode showed no fluctuation of the metal electrode after the SET process, which means VCM interface-type switching.

As presented in Figure 5A, Xu et al. verified the Ag migration inside the δ-CsPbI_3_ HP active layer between Ag electrodes through the top-view SEM.^84^ After applying a constant current to the memristor for 12 h, the growth of dendrite-like microstructure Ag metallic filaments was confirmed by SEM, while there was no change in SEM images when using Au electrodes, so it was concluded that the memristor was operated through Ag cation migration throughout the HP layer. Furthermore, Luo et al. observed the mechanisms in various ways (Figure 5B) through time-of-flight secondary ion mass spectrometry (ToF-SIMS), *in situ* PL imaging microscopy, EDS mapping on SEM image, and conducted the electrochemical impedance spectroscopy (EIS) to visualize the in-depth analysis of MAPbI_3_-based HP memristor.^85^ Wang et al. experimentally supported the hypothesis of synergetic ECM and VCM operation by fabrication of a CsPbBr_3_ quantum dot (QD)-based memristor with the ability to be driven by light illumination and comparing the Ag, Br, and Pb components’ ratio through energy dispersive X-ray spectroscopy (EDX) of field emission SEM (FE-SEM) in the lateral device.^50^ By analyzing the elemental distribution in the switching active layer in the LRS state, it was seen that the metal conductive filament and the halide anion vacancy filament were formed to cause the RS effect.

Xie et al. investigated GD-OES, SCLC, and EIS to unravel the operation mechanisms in RbPbI_3_ HP-based neuromorphic memristor devices combined with drift and diffusive modes.^45^ The switching mechanisms and ion migration/redistribution processes were explained by analyzing fluctuations in Ag, Sn, and I components after cyclic resistive switching and LTP/LTD synaptic simulation in the active bulk HP layer through GD-OES depth profiling and tracking the transformation of the complex IS according to the frequency. IS has actively contributed a great deal to analyzing the *I*–*V* hysteresis of HP-based solar cells, and in addition, several groups have recently attempted to analyze the operation mechanisms of HP-based memristors by connection impedance spectra with switching mechanism (Figure 5C,D) because IS can provide specific information on the switching inherent dynamic phenomena and responses of memristors and physical models.^78,89,90^ In Figure 5C, IS was used to demonstrate the dynamic state transition in 2D Ruddlesden–Popper HP-based memristor,^48,78^ where the evolution of the spectrum was analyzed according to the differentially applied switching voltages.^78^ The negative capacitance arc shape of the low-frequency arc, which is related to a slow kinetic phenomenon, was analyzed to determine the switching type (interface-type/filamentary-type) depending on the presence or absence of the Spiro-OMeTAD layer at the interface between the HP and the electrode where ionic migration and redistribution occur.

Following research on the dynamic state transition mechanism of IS and memristors, several physical models for memristive mechanism analysis have been proposed and give a new approach to understand the memristor physical dynamics, such as capacitive and inductive features.^90^ The switching mechanism can be analyzed by utilizing the actual dynamic operation models and time transient voltage response obtained from IS of the HP-based memristor, which shows complex ionic–electronic characteristics and also expanded to the HP-based neuromorphic networks (Figure 5D). IS is one of the characterization techniques that allows decoupling of physical processes with different time transient characterization at the memristive device operating conditions and gives a hint of what we want to improve in the circuit device. As such, various microscopic and spectroscopic observations on the driving operation mechanism of HP-based memristive devices become macroscopically insightful. Bisquert et al. explored the time domain and IS response of the HP-based memristor model with capacitive and inductive effects, especially focusing on the synaptic potentiation, and time-transient responses similar to the ion channel behavior in neurons.^86^ It has been shown that in the set process of an HP memristor underlies a chemical inductor mechanism,^91−94^ which explains the hysteresis effects^95^ and the time evolution of the resistance and the potentiation and depression evolution features in synaptic memristors.^96,97^

### 2.5 Various Efforts for the Desired Conductive Pathway Based on the Mechanism Analysis

**Figure 6:**
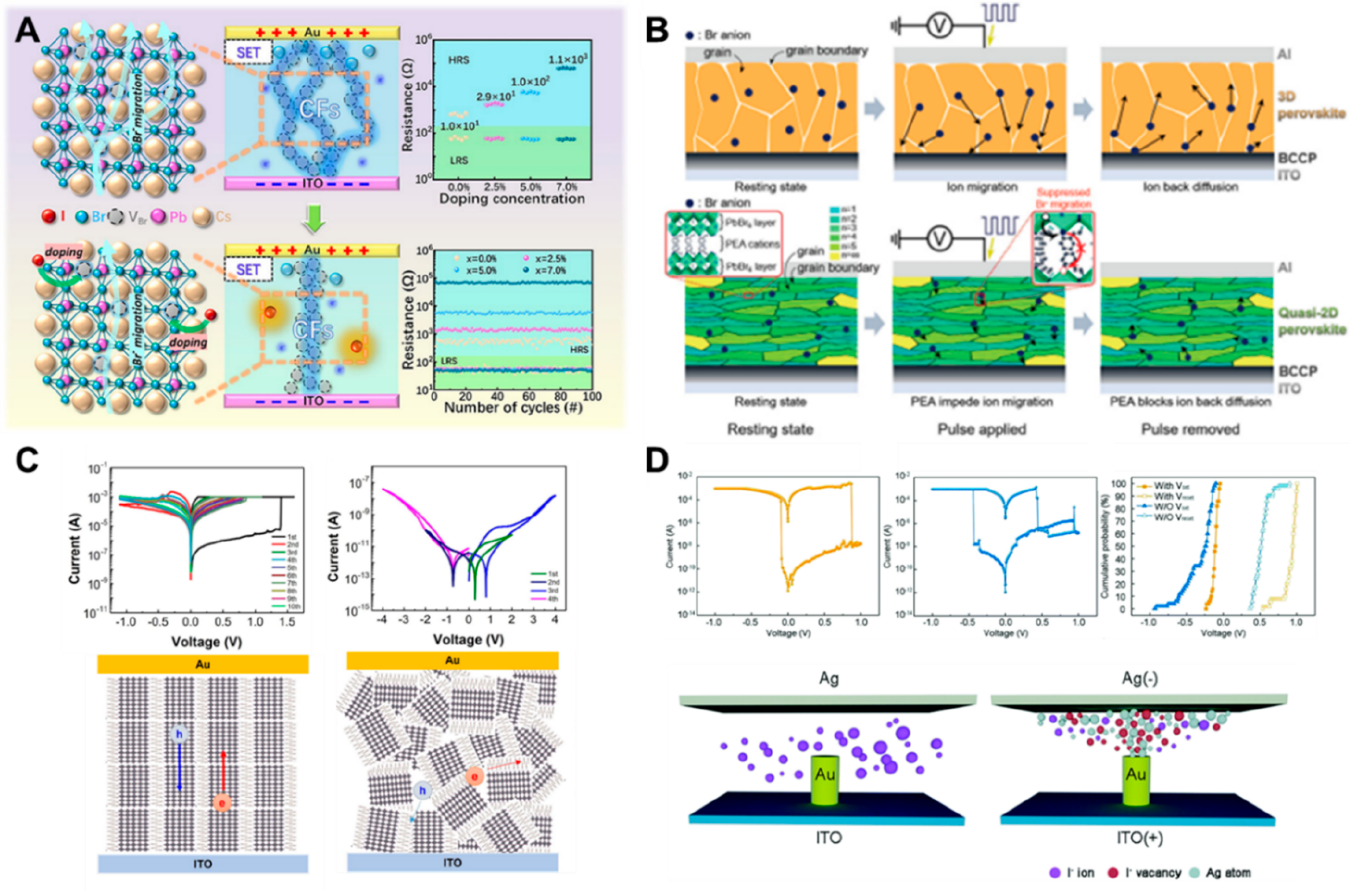
(A) Control of switching mechanism through halide ion doping. Reproduced from Li, A.; Wang, Z.; Peng, Z.; Zhou, J.; Chen, W. Improvement in Stability and Storage Performance of All-Inorganic Perovskite CsPb(Br_1–X_I_X_)_3_ Memristors Based on Simple Halide Ion Migration. *ACS Appl*. *Electron*. *Mater*. **2024**, 6, 3801.^38^ Copyright 2024 American Chemical Society. (B) Schematic diagram that operates differently in HP-based neuromorphic devices using the flexible dimensionality of HPs. Reproduced from Kim, S.-I.; Lee, Y.; Park, M.-H.; Go, G.-T.; Kim, Y.-H.; Xu, W.; Lee, H.-D.; Kim, H.; Seo, D.-G.; Lee, W.; Lee, T.-W. Dimensionality Dependent Plasticity in Halide Perovskite Artificial Synapses for Neuromorphic Computing. *Adv*. *Electron*. *Mater*. **2019**, 5 (9), 1900008.^22^ Copyright 2019 John Wiley and Sons. (C) Schematic RS mechanism that controls the movement and mechanism of the charge carriers by configuring the heterojunction-type device. Reproduced from Kim, S. J.; Lee, T. H.; Yang, J.-M.; Yang, J. W.; Lee, Y. J.; Choi, M.-J.; Lee, S. A.; Suh, J. M.; Kwak, K. J.; Baek, J. H.; Im, I. H.; Lee, D. E.; Kim, J. Y.; Kim, J.; Han, J. S.; Kim, S. Y.; Lee, D.; Park, N.-G.; Jang, H. W. Vertically Aligned Two-Dimensional Halide Perovskites for Reliably Operable Artificial Synapses. *Mater*. *Today***2022**, 52, 19.^60^ Copyright 2022 Elsevier. (D) Schematics of conductive mechanisms controlled using artificial Au tip on memristor. Reproduced from Chen, J.; Feng, Z.; Luo, M.; Wang, J.; Wang, Z.; Gong, Y.; Huang, S.; Qian, F.; Zhou, Y.; Han, S.-T. High-Performance Perovskite Memristor by Integrating a Tip-Shape Contact. *J. Mater. Chem. C***2021**, 9, 15435.^98^ Copyright 2021 Royal Society of Chemistry.

Based on the analyzed mechanisms, a variety of electrical/mechanical efforts have been proposed to reduce this randomness and control the working process in HP-based memristors, as shown in Figure 6. Li et al. controlled halide ion doping concentration for reducing the bromine vacancies on the surface film of all-inorganic CsPbX_3_ QD-based memristors (driven through the halide vacancy-composed filamentary type switching), where the memristive device can show improvement with more stable RS properties due to suppression of the randomly formed conductive channels (Figure 6A).^38^ Randomly spread vacancies due to Br ions with low mobility barriers indirectly were controlled by confining the area of CFs through the doping control of I ions, and the extent of abrupt switching changes of *I*–*V* curves was also changed by adjusting the resistivity in the device, where the defect formation energy of internal halide vacancy defects was calculated and provided through the dopant *ab initio* simulation package (DASP), and the defect was also detected by position annihilation spectrometry. As shown in Figure 6B, the ion migration mechanism of a neuromorphic device can be controlled by utilizing the flexible dimensionality of HPs. Phenylethylammonium ion (PEA) was inserted from 3D structured MAPbBr_3_ film to induce quasi-2D (PEA)_2_(MA)_*n*−2_Pb_*n*_Br_3*n*+1_ in HP-based artificial synapses, where the synaptic plasticity was transformed by controlling ion migration and diffusion.^22^

Kim et al. successfully obtained the vertically aligned ion transport channel by utilizing vertically aligned 2D HPs in an artificial synapse in view of HP nanophysics, proved by first-principles calculations (Figure 6C).^60^ Compared to the random-oriented HP-based memristor (right -hand panel) which showed no electrical postsynaptic potential responses, the vertically aligned HP-based memristor (left-hand panel) can be well-controlled to the linear conductance change with synaptic plasticity. To artificially guide the relatively complex ionic–electronic movement of HPs in a preferred direction from a mechanical point of view, Chen et al. successfully controlled the growth of CFs (composed of Ag atoms and I vacancies) in the filamentary-type switching by establishing a 200 nm thick Au tip on the bottom electrode of ITO through e-beam photolithography and observed the device operation with cross-sectional TEM and EELS mapping (Figure 6D).^98^ In addition, Wu’s group transformed the trapping/detrapping conduction mechanism of the injected carriers by internal inherent defects from the perspective of the conduction mechanism through Ag filament by using the heterojunction generated at the interface through the integrating architecture of multiple semiconductors with HPs, and they investigated the mechanisms and performance of the HP-based memristors with a single layer and an integrated double layer.^99^

Like these various methods and efforts, the most important thing is to accurately identify and control the operating mechanism principles for solving these problems and to achieve higher performance and stable device implementation. While the various approaches for mechanism analysis described here contribute significantly to the development of HP-based neuromorphic devices and memristors, it is true that HPs still have complex and abundant mechanisms that cannot be anticipated and can hinder enhanced development.

In summary, the advancements of HP-based two-terminal memristors’ operating mechanisms and principles have been reviewed here, which are essential for reaching higher performances and enabling appropriate multifunctional memristive applications. HP-based memristors and their neuromorphic devices have advanced rapidly in terms of performance and application through various experimental efforts, but now fundamental mechanism analysis has become essential to break the limits and go further. Since the widespread mechanism proposals and analyses can make further research troublesome, we here categorized and summarized the memristive switching origins and advancements, focusing on the efforts to clarify the mechanisms.

Focusing on the operating mechanisms, we simply showed the leading HP-based memristive devices and explored the driving principles that cause their superior characteristics. We categorized the mechanism explanations with summary tables to simplify the presentation with representative reports categorized by switching type (filamentary type/interface type), switching mechanisms based on the ionic species type of conductive pathway (ECM/VCM), switching motion type (abrupt/gradual or digital/analog switching), electrical and physical analysis for understanding the switching mechanisms, and various efforts for the desired driving operation with mechanism analysis. As a result of the many efforts for mechanism analysis to date, the operation principles of HP-based memristors and neuromorphic devices are being identified, but complex intrinsic HP-switching dynamics and syergetic analysis from circuit-, chemistry-, and physics-based perspectives are still insufficient. In order to properly utilize the unique *I*–*V* hysteresis characteristics of HPs as multifunctional memristors, for use in devices from integrated memory circuits to bioinspired memristors such as artificial neurons/synapses, the intended ionic/electronic movements in the devices should be the result of a fine-tuning strategy. As many previous studies have shown, since all the components of the memristive devices can affect the switching mechanisms and operation progress, understanding the switching mechanism/principle and the role of each component from various perspectives is the key to further progress.
